# Complete genome sequences of *Pantoea vagans* strains GM1 and GM2, isolated from the leaves of garlic mustard

**DOI:** 10.1128/mra.00943-24

**Published:** 2024-12-10

**Authors:** Gi Yoon Shin, Brian H. Kvitko

**Affiliations:** 1 Department of Plant Pathology, University of Georgia, Athens, Georgia, USA; Rochester Institute of Technology, Rochester, New York, USA

**Keywords:** *Pantoea*, HiVir, phosphonate, phytotoxin, garlic mustard, *Alliaria*

## Abstract

We report the complete genomes of two *Pantoea vagans* strains, GM1 and GM2, isolated from garlic mustard plants. *P. vagans* strain GM1 was found to carry a HiVir pantaphos biosynthetic gene cluster, which causes onion necrosis.

## ANNOUNCEMENT

Many *Pantoea* species are agriculturally significant. The HiVir biosynthetic gene cluster (BGC), which produces necrosis-inducing phosphonate toxin pantaphos (1), is a key virulence factor in *Pantoea* species causing Onion Center Rot (2–6). Here, we report the complete genomes of *Pantoea vagans* GM1 and GM2, isolated from asymptomatic *Alliaria petiolata* L. (garlic mustard) leaves. Notably, *P. vagans* GM1 encodes the HiVir BGC and induces necrosis on onion scales.

Invasive garlic mustard plants were removed from the “Second Saddle” population near Pigeon Hill Trail, Kennesaw Mountain National Battlefield Park, Marietta, GA (GPS: 33.967062–84.587307) on 10/28/2022. Approximately 5 g of leaf tissue was hand-macerated in a zip-top bag with 5 mL of sterile 0.25 mM MgCl_2_. After 10 minutes at room temperature, 0.2 mL volume of leaf wash was plated onto Lysogeny Broth (LB) agar +200 µg/mL cycloheximide and incubated at 37°C for 1 day. Yellow colonies were streaked to isolation on LB agar and stored as 15% glycerol cryostocks at −80°C and tested for red onion scale necrosis as per Shin et al. (6).

Genomic extraction and sequencing were conducted by Plasmidsaurus (Eugene, OR, USA). DNA was extracted from 1 mL cell pellets of 24-hour LB culture shipped in a DNA/RNA shield using Quick-DNA Fungal/Bacteria Miniprep Kit (Zymo Research, CA, USA). Libraries were prepared with ONT Rapid Barcoding Kit96 v14 (SQK-RBK114.96) without ligation, shearing, or size selection and sequenced on PromethION P24 with R10.4.1 flow cells, generating 72,075 reads (rN50 11,038 bp) for GM1 and 80,693 reads (rN50 10,927 bp) for GM2. Base calling was performed with ont-doradod-for-promethion v7.1.4 on super accurate mode. Filtlong v0.2.1 (https://github.com/rrwick/Filtlong) removed the low-quality reads and the remaining reads were assembled and polished with Flye v2.9.1 (7) and Medaka v1.9.0 (https://github.com/nanoporetech/medaka). Contig analysis was performed using Bandage v0.8.1 (8), and genome quality was assessed with Quast v5.2.0 (9) and CheckM v1.2.2 (10). Digital DNA-DNA hybridization (dDDH) (https://tygs.dsmz.de/) and average nucleotide identity (ANI) using the OrthoANI tool v0.93.1 (11) were conducted for taxonomic identification. HiVir BGC was identified using BLAST+v2.14.1 (12) search with PNA97-1 HiVir BGC (NZ_CP020943.2, coordinates: 833001–845532). Assemblies were annotated using the NCBI PGAP v6.8. Default parameters were used for all software except where otherwise noted.

**Fig 1:**
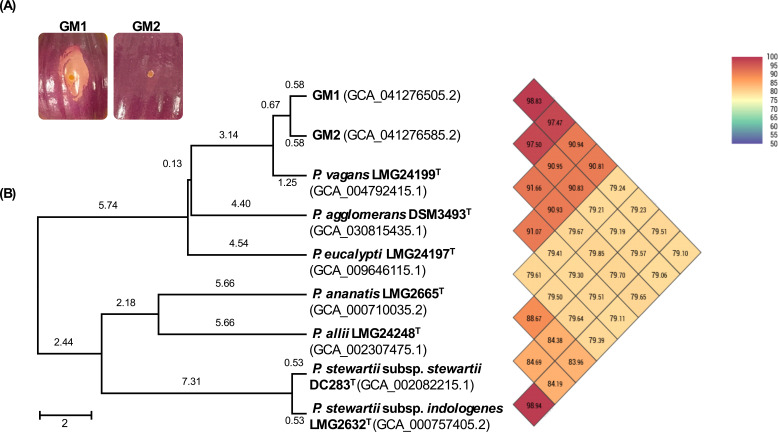
(**A**) HiVir-positive GM1 causes necrosis on detached red onion scales, whereas HiVir-negative GM2 does not. (**B**) GM1 and GM2 are identified as *P. vagans*, with over 97% ANI values to *P. vagans* LMG24199 ^T^.

GM1 genome assembly comprised four contigs (total 410,576,250 bp, N50 of 4,061,763 bp, 4,632 genes) and GM2 five contigs (total 453,128,947 bp, N50 of 4,003,966 bp, 4,636 genes), with assembled coverages of 80× and 88×, respectively. Both assemblies had a GC content of 55% and shared an 83% pairwise dDDH (Table 1) and 97.5% ANI with the type strain of *P. vagans* LMG 24199^T^ (Fig. 1). GM1 plasmid (549,416 bp) encoded a HiVir BGC that is 87% identical with that of PNA97-1 although lacking *hvrK* which is dispensable for HiVir-mediated necrosis (4).

**TABLE 1:** Pairwise dDDH values of GM1 and GM2 against *Pantoea* spp

Query strain	Subject strain (GenBank accession)	dDDH (%)
GM1	GM2	93.7
GM1	*P. vagans* LMG24199^T^ (GCA_004792415.1)	83.6
GM1	*P. agglomerans* DSM3493 ^T^ (GCA_030815435.1)	80.7
GM1	*P. eucalypti* LMG24197 ^T^ (GCA_009646115.1)	71.6
GM1	*P. deleyi* LMG24200 ^T^ (GCA_022647325.1)	64.5
GM1	*P. anthophila* LMG2558 ^T^ (GCA_006494375.1)	70.6
GM1	*P. conspicua* LMG24534 ^T^ (GCA_002095315.1)	60.1
GM1	*P. brenneri* LMG5343 T (GCA_002095575.1)	59.9
GM1	*P. ananatis* LMG2665 ^T^ (GCA_000710035.2)	31.4
GM2	*P. vagans* LMG24199 ^T^ (GCA_004792415.1)	83.7
GM2	*P. agglomerans* DSM3493 ^T^ (GCA_030815435.1)	79
GM2	*P. eucalypti* LMG24197 ^T^ (GCA_009646115.1)	71.8
GM2	*P. deleyi* LMG24200 ^T^ (GCA_022647325.1)	64.8
GM2	*P. anthophila* LMG2558 ^T^ (GCA_006494375.1)	70.2
GM2	*P. conspicua* LMG24534 ^T^ (GCA_002095315.1)	59.7
GM2	*P. brenneri* LMG5343 ^T^ (GCA_002095575.1)	60.8
GM2	*P. ananatis* LMG2665 ^T^ (GCA_000710035.2)	31.1

## Data Availability

The whole-genome sequences and raw reads of *P. vagans* GM1 and GM2 strains have been deposited with NCBI GenBank under the project number of PRJNA1069770. The accession numbers are GCA_041276505.2 and GCA_041276585.2 for genome assemblies and SRX25923284 and SRX25923285 for raw reads of GM1 and GM2, respectively.
